# Supplementary material for: Bayesian refinement of protein structures and ensembles against SAXS data using molecular dynamics
Source: PLoS Comput Biol. 2017 Oct 18;13(10):e1005800. doi: 10.1371/journal.pcbi.1005800 (PMC5662244; doi:10.1371/journal.pcbi.1005800)
Supplement: S1 Archive — (BZ2) [file pcbi.1005800.s011.tar.bz2 › gromacs_4.62_bayesian_saxs_refi/share/top/charmm22star.ff/forcefield.doc]

CHARMM22* all-atom force field

*******************************************************************************

*	CHARMM port writted by	*

*	Par Bjelkmar, Per Larsson, Michel Cuendet, *

*	Berk Hess and Erik Lindahl. *

*	Correspondance: *

*	bjelkmar@cbr.su.se or lindahl@cbr.su.se	*

*	CHARMM22* modifications implemented by	*

*	Karl Debiec	*

*	Correspondance: *

*	ktd3@pitt.edu	*

*******************************************************************************

Parameters derived from c32b1 version of CHARMM

NOTE: Atom-based charge groups

References:

Proteins

MacKerell, Jr., A. D., Feig, M., Brooks, C.L., III, Extending the

treatment of backbone energetics in protein force fields: limitations

of gas-phase quantum mechanics in reproducing protein conformational

distributions in molecular dynamics simulations, Journal of

Computational Chemistry, 25: 1400-1415, 2004.

and

MacKerell, Jr., A. D., et al. All-atom

empirical potential for molecular modeling and dynamics Studies of

proteins. Journal of Physical Chemistry B, 1998, 102, 3586-3616.

and

Piana, S., et al. How robust are protein-folding simulations with

respect to force field parameterization?, Biophysical Journal, 100:

L47-L49, 2011.

Lipids

Feller, S. and MacKerell, Jr., A.D. An Improved Empirical Potential

Energy Function for Molecular Simulations of Phospholipids, Journal

of Physical Chemistry B, 2000, 104: 7510-7515.

Nucleic Acids

Foloppe, N. and MacKerell, Jr., A.D. "All-Atom Empirical Force Field for

Nucleic Acids: 2) Parameter Optimization Based on Small Molecule and

Condensed Phase Macromolecular Target Data. 2000, 21: 86-104.

and

MacKerell, Jr., A.D. and Banavali, N. "All-Atom Empirical Force Field for

Nucleic Acids: 2) Application to Molecular Dynamics Simulations of DNA

and RNA in Solution. 2000, 21: 105-120.

If using there parameters for research please cite:

Bjelkmar, P., Larsson, P., Cuendet, M. A, Bess, B., Lindahl, E.

Implementation of the CHARMM force field in GROMACS: Analysis of protein

stability effects from correction maps, virtual interaction sites, and

water models., Journal of Chemical Theory and Computation, 6: 459-466, 2010.

and

Piana, S., Lindorff-Larsen, K., Shaw, D. E. How robust are protein-folding

simulations with respect to force field parameterization?.,

Biophysical Journal, 100: L47-L49, 2011.
